# The Mildmay Mission Hospital

**Published:** 1887-02-19

**Authors:** 


### THE MILDMAY MISSION HOSPITAL.

By a Young Hand.

The work of the Mildmay Mission in various parts of our great metropolis is too widely distributed, and has done too much good work, to be unknown to any of our readers. It is, however, of its hospital at Bethnal Green, and its medical work in the East End, that we propose to give some short account in the present notice. The hospital buildings just now are somewhat inefficient, being adapted, so far as it has been found possible to do so, from an old factory, which is leased at the high rental of £350 per annum. This lease will expire in the course of the current year, and it is for many reasons considered undesirable to renew the tenancy for a further period. Turville Street, in which the hospital is situated, is a great thoroughfare, and the noise from the constant traffic, as well as the immediate vicinity of a public-house, with the blasphemous language and noisy quarrels of the denizens of the quarter, render it a very undesirable position for an institution whose object is the care and cure of the sick. The same causes are found detrimental to the health of the nurses and attendants, who suffer both by day and night from the continual uproar and interruption of their necessary rest. The management, therefore, feel that it is most desirable to secure a suitable building in a quieter locality, and one whose arrangements are better adapted to the needs of a hospital. There is at present a children's ward with twelve cots, and as these are nearly always full, increased accommodation in this department alone is much required.

The homes to which these children have to return furnish food for much sad reflection. In proof of the melancholy conditions under which many of them grow up, we may incidentally quote the following anecdote from the Report of the hospital. A curly-headed, bright-eyed boy in the ward was asked what his father did, referring to his occupation. The answer was, "Beats mother." And when again asked what he did when not beating mother, he said, "He mends shoes." The daily visiting of patients in their own homes (for, as is usual in most of our London hospitals, the out-patient department is a very important feature of the work) by the doctors and nurses, affords valuable opportunities for diffusing a good and wholesome influence amongst the families who live in the crowded and squalid dwellings of the vicinity. That such opportunities are not neglected by the workers for the Mildmay Mission may be taken for granted, and many interesting accounts of the good done in the promotion of religious feeling are given in the report.

The Society has a Convalescent Home at Barnet, which is supported by Lord and Lady Tankerville, and has proved of incalculable value to many of the patients who have enjoyed its benefits. It is stated that no less than 450 men, women, and children have visited this Home since its establishment. The soup-kitchens of the Mission at Bethnal Green have been altered and enlarged, so that they now supply a greater number of people than was formerly the case; and the customers for dinners of soup and bread at the price of one penny per portion are numerous. Gifts of blankets, sheets, and garments of any description, old or new, are always gratefully received by the Mission; whilst flannel, books, toys, empty bottles, etc., for distribution to the more needy of the dwellers in the neighbourhood, are most acceptable. Much illness is caused through insufficient protection from cold and damp, and clothing for the use of convalescent patients is always in request. One poor woman, a rheumatic patient, who was discharged cured from the hospital, had only two ragged skirts, an old dress body, a piece of an old shawl, a bonnet, and a pair of broken boots to go away in. With such meagre clothing her future immunity from rheumatism could not be hoped for, and it is in such cases that the gifts of old garments from friends of the institution prove so valuable. Any gifts for the sick of the district should be marked "Medical Mission"; and those for the general poor, "Mildmay Mission House." All contributions to be sent direct to the Mildmay Mission, 82, Church Street, Bethnal Green, E.

